# Impairment of the Bacterial Biofilm Stability by Triclosan

**DOI:** 10.1371/journal.pone.0031183

**Published:** 2012-04-16

**Authors:** Helen V. Lubarsky, Sabine U. Gerbersdorf, Cédric Hubas, Sebastian Behrens, Francesco Ricciardi, David M. Paterson

**Affiliations:** 1 Institute of Hydraulic Engineering, University Stuttgart, Stuttgart, Germany; 2 Sediment Ecology Research Group, Scottish Ocean Institute, School of Biology, University of St. Andrews, St. Andrews, Scotland, United Kingdom; 3 Département Milieux et Peuplements Aquatiques (DMPA), Muséum National d’Histoire Naturelle, UMR BOREA (Biologie des organismes et écosystèmes aquatiques) MNHN-CNRS-UPMC-IRD, Paris, France; 4 Geomicrobiology/Microbial Ecology Group, Centre for Applied Geosciences (ZAG), Eberhard-Karls-University Tübingen, Tübingen, Germany; 5 Institute of Aquatic Ecology, University of Girona, Girona, Spain; East Carolina University School of Medicine, United States of America

## Abstract

The accumulation of the widely-used antibacterial and antifungal compound triclosan (TCS) in freshwaters raises concerns about the impact of this harmful chemical on the biofilms that are the dominant life style of microorganisms in aquatic systems. However, investigations to-date rarely go beyond effects at the cellular, physiological or morphological level. The present paper focuses on bacterial biofilms addressing the possible chemical impairment of their functionality, while also examining their substratum stabilization potential as one example of an important ecosystem service. The development of a bacterial assemblage of natural composition – isolated from sediments of the Eden Estuary (Scotland, UK) – on non-cohesive glass beads (<63 µm) and exposed to a range of triclosan concentrations (control, 2 – 100 µg L^−1^) was monitored over time by Magnetic Particle Induction (MagPI). In parallel, bacterial cell numbers, division rate, community composition (DGGE) and EPS (extracellular polymeric substances: carbohydrates and proteins) secretion were determined. While the triclosan exposure did not prevent bacterial settlement, biofilm development was increasingly inhibited by increasing TCS levels. The surface binding capacity (MagPI) of the assemblages was positively correlated to the microbial secreted EPS matrix. The EPS concentrations and composition (quantity and quality) were closely linked to bacterial growth, which was affected by enhanced TCS exposure. Furthermore, TCS induced significant changes in bacterial community composition as well as a significant decrease in bacterial diversity. The impairment of the stabilization potential of bacterial biofilm under even low, environmentally relevant TCS levels is of concern since the resistance of sediments to erosive forces has large implications for the dynamics of sediments and associated pollutant dispersal. In addition, the surface adhesive capacity of the biofilm acts as a sensitive measure of ecosystem effects.

## Introduction

### Triclosan – a Recent Chemical in Aquatic Habitats

Triclosan (5-chloro-2-(2,4-dichlorophenoxy)phenol), also known as irgasan, is a broad-spectrum antibacterial and antifungal compound that has been widely used since the 1970s in pharmaceutical personal care products (PPCPs), textiles, cleaning supplies, toys and computer equipment [1]. About 96% of triclosan (TCS) originating from consumer products is discarded in residential drains [2], leading to considerable loads of the chemical in waters entering wastewater treatment plants (WWTP). While biological sewage treatment had been regarded as an effective barrier for TCS due to removal efficiencies of 98% in the aqueous phase, Heidler & Halden [3] showed that the particle-associated TCS was sequestered into waste-water residuals and accumulated in the sludge with less than half of the total mass being bio-transformed or lost. Consequently, substantial quantities of the chemical can be transferred into soils and groundwater by sludge recycling [3] or directly enters rivers with estimated concentrations usually between 11 – 98 ng/L [1] but with up to 2.7 µg/L [4] recorded. In the aqueous phase, the transformation of TCS into a variety of polychlorinated dibenzo-p-dioxins under the exposure of sunlight and especially at high pH values becomes problematic; the levels of the four main dioxins derived from triclosan have risen by 200 to 300% in the last 30 years [5]. Although there is evidence that TCS is readily biodegradable under aerobic conditions in the water column [6], TCS is still regarded as one of the top 10 of persistent contaminants in U.S. rivers, streams, lakes, and underground aquifers due to its continuous replenishment and its accumulation within the sediments [7], [8]. Increasing TCS concentrations have been reported world-wide from many countries for rivers, lakes and streams, being currently in the range of 18 ng/L – 2.7 µg/L in the water column [1], [4], [7], [9] while 0.27 to 130.7 µg/kg TCS have been determined in sediments [10], [11].

### Triclosan – Mode of Action

Triclosan was originally introduced as a non-specific biocide but has been shown to affect bacterial membranes as a consequence of the specific inhibition of the fatty acid biosynthesis [12]. TCS specifically inhibits the enzyme enoyl-acyl carrier protein reductase (ENR) FabI by mimicking its natural substrate, thus blocking the final, regulatory step in the fatty-acid synthesis cycle [13]. Consequently, bacterial cells can acquire resistance versus TCS from missense mutations in the *fabI* gene; as has been shown for several strains of *Escherichia coli* [14], [15]. Triclosan also caused up-regulated the transcription of other genes (e.g. micF, acrAB, marA bcsA, bcsE) in *Salmonella* that might help induce further resistance [16]. Schweizer [17] reported that some bacterial strains (such as gram-negative bacteria) use a multiple triclosan resistance mechanism, including active efflux from cell where bacteria actively pump TCS out of the cell [18]. Moreover, some bacteria have been shown to produce triclosan-insusceptible enzymes [19] or triclosan-degradative enzymes [20] and also the capability to modify the outer membrane permeability barriers [21]. Although it has been investigated whether the inhibition of the metabolic pathway via ENR can solely explain the complex mode of action and lethality of TCS for bacteria [15], other impairments of bacterial functions by TCS have not yet been established. Moreover, there is little information on possible shifts within the bacterial community due to TCS exposure, or the consequences of genetic modifications for environmental bacterial functionality [22].

### Triclosan – More than Just Concentration

The effects of TCS on bacteria may vary according to the concentration of the chemical, its bioavailability, the exposure time, the physiology of the target organisms and the targeted species. For instance, Russell [21] reported that TCS affects many, but not all, types of Gram-positive and Gram-negative bacteria. Inactive bacteria seem to be more resilient to the lethal effects of TCS possibly due to a reduced metabolism and an enhanced physical barrier against TCS created by debris and dead cells in the stationary growth phase [23]. Low TCS concentrations (0.02 – 0.5 µg ml^−1^) affected the growth of several bacteria while higher TCS concentrations (5 – 50 mg l^−1^) were bactericidal regardless of the growth phase [23]. At higher concentrations, TCS seems to act rapidly and with highly damaging effects to multiple cytoplasmic and membrane targets, resulting in leakage of intracellular material [24]. However, in natural samples, lethal effects of TCS were observed, by using the bioluminescence assay of *Vibrio fisheri*, at much lower concentrations of environmental relevance. For instance, DeLorenzo et al. [25] reported an EC_50_ of 53 µg l^−1^ for estuarine samples and Farré et al. [26] determined an EC_50_ of 280 µg l^−1^ in waste-waters while Ricart et al. [27] observed mortality within a river biofilm at only 0.21 µg l^−1^ TCS. The same is true for the acute toxic effects of TCS exposure on co-occurring non-target components, especially for microalgae [22], [27], [28] and for higher organisms such as shrimps [29]. This indicates that the relatively low TCS concentrations currently measured in the aquatic habitats might have a profound effect on the resident organisms.

### Does TCS Impair Biostabilization by Bacterial Biofilms?

Despite numerous recent studies recognizing that TCS affects the growth, biomass, mortality and physiology of bacteria [17], [27], little is known about chronic effects (e.g. genotoxicity, mutagenicity) caused by long-term exposure. Much too rarely, research also includes important measures such as the architecture of biofilms as well as community shifts, although both might have a profound effect on the functionality of the microbial ecosystems [22]. There is no literature relating TCS exposure to the impairment of biofilm functionality despite biofilms representing the dominant microbial life forms in aquatic habitats that drive provisioning (e.g. food, clean drinking water), regulating (e.g. carbon sequestration, self-purification) and supporting (e.g. biogeochemical fluxes) services for their habitat and beyond [30]. One interesting ecosystem function or service is biostabilization where the microorganisms modify the response of aquatic sediments to erosive forces (flow velocity, turbulence) by the secretion of extracellular polymeric substances (EPS) [31]. In this context, EPS acts like a glue to bind the sediment grains together. Much more work has been published on microalgal rather than bacterial sediment stabilization [31], but recently the role of bacterial stabilization has been confirmed [32], [33], [34]. The present paper focuses on bacteria, since these microbes a). play a crucial role in biostabilization, b). are the primary target for TCS, and c). often dominate sediment surface biofilms in rivers and coastal areas devoid of light. Pollutants such as TCS might affect the functionality of biofilms by inducing shifts in species composition, affecting physiology of the tolerant species and thus impact EPS quantity and quality. Effects on EPS secretion due to pollutant exposure have been reported, ranging from elevated levels of EPS as a protective mechanisms of cells [35] to reduced EPS concentrations due to limited growth and metabolism [36]. Since the EPS matrix also offers a multitude of adsorption sites for pollutants to decrease their bioavailability and to bring them into close proximity to potential degraders, a reduction in EPS quantity might severely affect this biofilm function. If the stabilization of sediments by biofilms was decreased after pollutant exposure, sediment-bound pollutants might be more easily eroded to become bioavailable again; a classical negative feedback mechanism that has not been addressed so far.

### The Objectives of the Present Study

Knowledge on the biostabilization capacity of biofilms and their impairment by pollutant exposure is of high significance for sediment management strategies in waterways and coastal regions. The present study is a first step towards the investigation of the effects of triclosan on the stabilization potential of biofilms while focusing on natural bacterial assemblages exposed to different TCS concentrations (ranging from 2 – 100 µg/L). The lower TCS concentrations are within the range of values determined presently in the natural waters while medium and higher TCS concentrations were chosen to account for the known accumulation rates of TCS in sediments as well as for possible future scenarios when considering an ongoing continuous release of triclosan into the aquatic habitats. Over the course of 2 weeks, the adhesive capacity of the test surface, a proxy for sediment stability, was determined with a newly developed device (Magnetic Particle Induction MagPI, [37]). In parallel, bacterial cell numbers, division rates, species composition and EPS (proteins, carbohydrates) secretion were monitored and related to the adhesive capacity of the developing biofilms.

## Results

### Triclosan Concentrations

The actual triclosan concentrations within the substratum were about two times higher than the predicted concentrations (predicted  =  2 µg – 100 µg/L, actual  =  4 µg – 180 µg/L, from the lowest to the highest value). The actual triclosan concentrations in the overlying water were also two times higher than the predicted concentrations in the low range (predicted: 2 µg/L, actual: up to 4 µg/l), but were similar in predicted and actual values for the spiking concentrations in the medium (e.g. predicted: 50 µg/L, actual: 49 µg/l). Over the experimental period, some of the water within the glass tanks evaporated, but the total TCS concentrations in the water column and in the substratum did not change significantly over time (data not shown).

### The Stability of the Substratum

**Figure 1:**
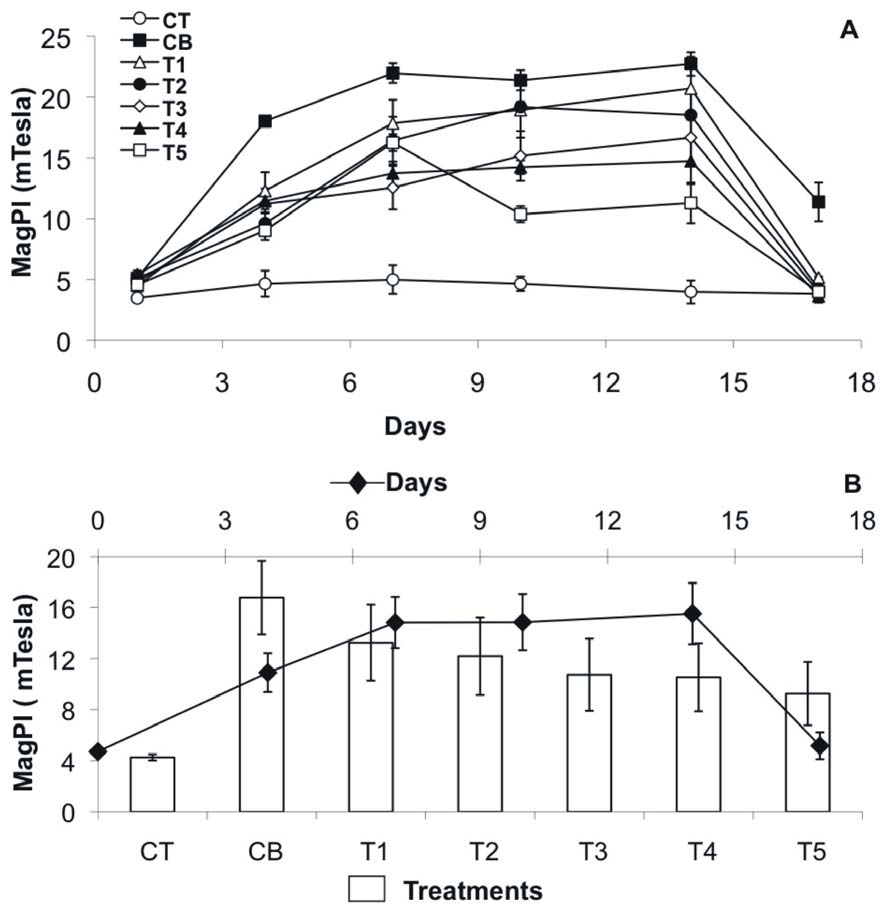
Biofilm adhesion as a proxy for stability, measured by MagPI, over the course of the experiment. (A) Mean values (n = 4 per treatment) with SE of the different treatments over time: positive control CB (black squares), negative control CT (white circles), T1 (TCS: 2 µg/L, white triangles), T2 (TCS: 10 µg/L, black circles), T3 (TCS: 20 µg/L, white diamonds), T4 (TCS: 50 µg/L, black triangle), T5 (TCS: 100 µg/L, white squares). (B) Mean values with SE per day (n = 7, black diamonds) and per treatment (n = 6, bar plots).

The adhesion of the substratum surface increased continuously in all treatments with biofilms up to day 14 and decreased afterwards (Fig. 1 A, B). In contrast, the negative control (CT) did not show any significant changes in adhesion over the experimental period (Fig. 1 A, B). In comparison to the negative control, the stability increases caused by the bacterial biofilms were most pronounced for the bacterial control CB and treatment T1 (up to 4.6 times) followed by T2 and T3 (up to 3.6) as well as T4 and T5 (up to 2.7) (Fig. 1 A, B, Table 1). Accordingly, the positive control without triclosan showed the highest surface adhesion of the sediment (CB) (22.73±1 mTesla), which then declined in the bacterial cultures with increasing TCS exposure: T1 (20.7±2.6 mTesla) > T2 (18.53±1.9 mTesla) > T3 (16.7±2.1 mTesla) > T4 (14.7±1.9 mTesla) > T5 (11.3±1.7 mTesla). The daily differences between the treatments were generally significant. For example on day 14, the stability of the biofilm without TCS (CB) was significantly higher than T3, T4, and T5 (Permanova p <0.0001, followed by a non-parametric SNK test).

**Table 1:** Ratios between variables.

	*Treatment*	*Adhesion -MagPI*	*EPS Carbohydrates*	*EPS Proteins*	*Bacterial cells*	*Bacterial division*
***Ratio A***	CB	4.4	3.5	2.2	2.0	8.3
	T1	4.6	3.2	1.4	1.9	1.9
	T2	3.6	2.6	1.8	1.4	3.1
	T3	3.5	1.7	1.6	1.5	1.1
	T4	2.7	2.9	1.8	1.2	4.0
	T5	2.5	3.5	1.1	1.7	3.7
***Ratio B***	T1	1.1	1.3	1.4	0.9	1.4
	T2	1.2	1.4	1.4	1.5	1.2
	T3	1.3	1.8	1.7	1.4	1.3
	T4	1.5	1.1	1.0	2.5	1.0
	T5	2.0	1.6	1.3	1.9	1.0

A. Ratio for different variables between the first day (start) and day 14 (end) of the experiment. B. Ratio for different variables between the positive control “CB” and the treatments (“T1, T2, T3, T4, T5”).

### Bacterial Cell Numbers and Growth Rate

**Figure 2:**
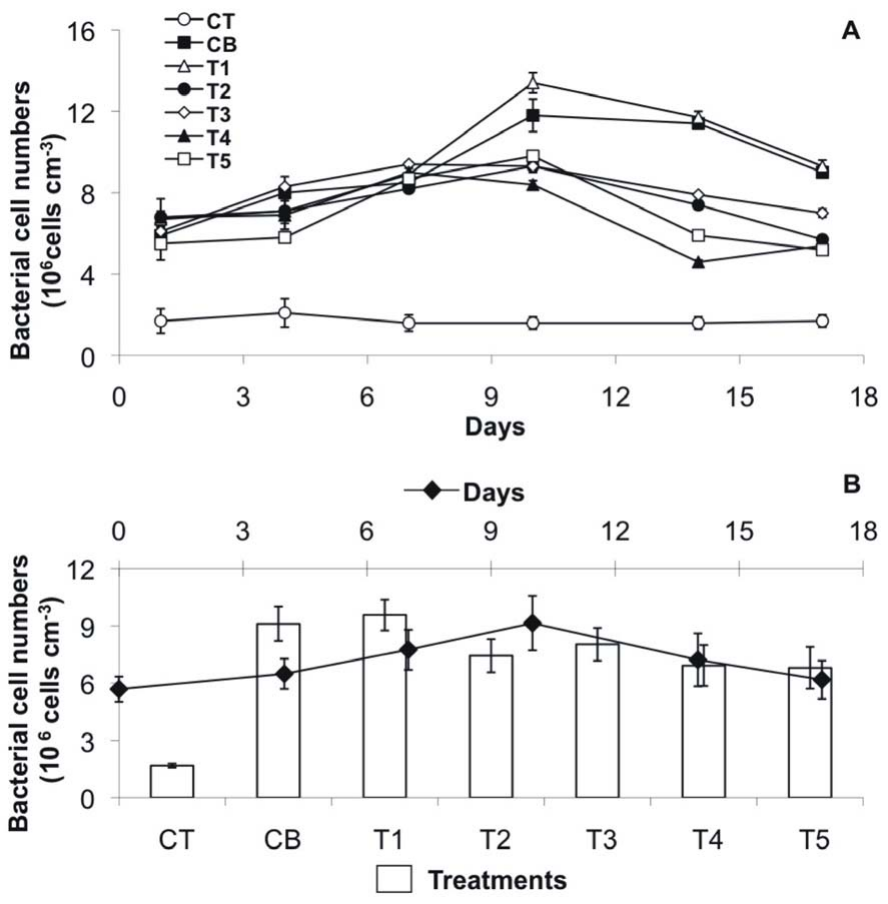
Bacterial cell numbers over the course of the experiment. (A) Mean values (n = 4 per treatment) with SE of the different treatments over time: positive control CB (black squares), negative control CT (white circles), T1 (TCS: 2 µg/L, white triangles), T2 (TCS: 10 µg/L, black circles), T3 (TCS: 20 µg/L, white diamonds), T4 (TCS: 50 µg/L, black triangle), T5 (TCS: 100 µg/L, white squares). (B) Mean values with SE per day (n = 7, black diamonds) and per treatment (n = 6, bar plots).

In the first experimental week, the bacterial cell numbers increased in all treatments up to day 10 (Fig. 2A, B). The increase was more pronounced for the treatments CB and T1 (up to 2) with bacterial cell numbers ranging from 5.9×10^6^ to 12×10^6^ cells cm^−3^ and 6.7×10^6^ to 13×10^6^ cells cm^−3^, respectively (Fig. 2 A, Table 1). Generally, the other treatments showed significantly lower bacterial cell numbers. The daily differences between the treatments were generally significant (Permanova). For example, on day 14, both treatments CB and T1 were significantly higher in bacterial cell number than T4 and T5 (Permanova, p<0.0001, followed by a non-parametric SNK test). A general decrease of bacterial cell numbers along with increasing TCS concentrations was observed, except for T1, which was quite similar to the positive control (Fig. 2 B).

The bacterial division rates of the community were highly variable within the treatments over time (Table 2). However, the bacterial biofilm without triclosan (CB) showed a more consistent and pronounced increase in the bacterial division rates with time as compared to the TCS treatments (Table 1). No significant relations could be determined between bacterial cell numbers and bacterial division rates in the different treatments. As for the bacterial cell numbers, the bacterial division rates were negligible in the negative controls.

**Table 2:** Bacterial dividing rates in treatments over the experimental time (10^6^ cells cm^−3^ h^−1^).

	*Day 1*	*Day 2*	*Day 3*	*Day 4*	*Day 5*	*Day 6*
CB	0.64	2.30	5.13	3.48	5.33	1.53
T1	2.04	0.91	0.24	1.37	3.89	1.47
T2	1.41	4.14	2.77	4.03	4.46	1.30
T3	3.81	4.23	2.72	2.85	4.01	1.32
T4	2.06	2.91	8.43	2.76	4.86	1.46
T5	1.33	0.11	4.61	3.72	4.95	0.64

### Bacterial Community Composition

**Figure 3:**
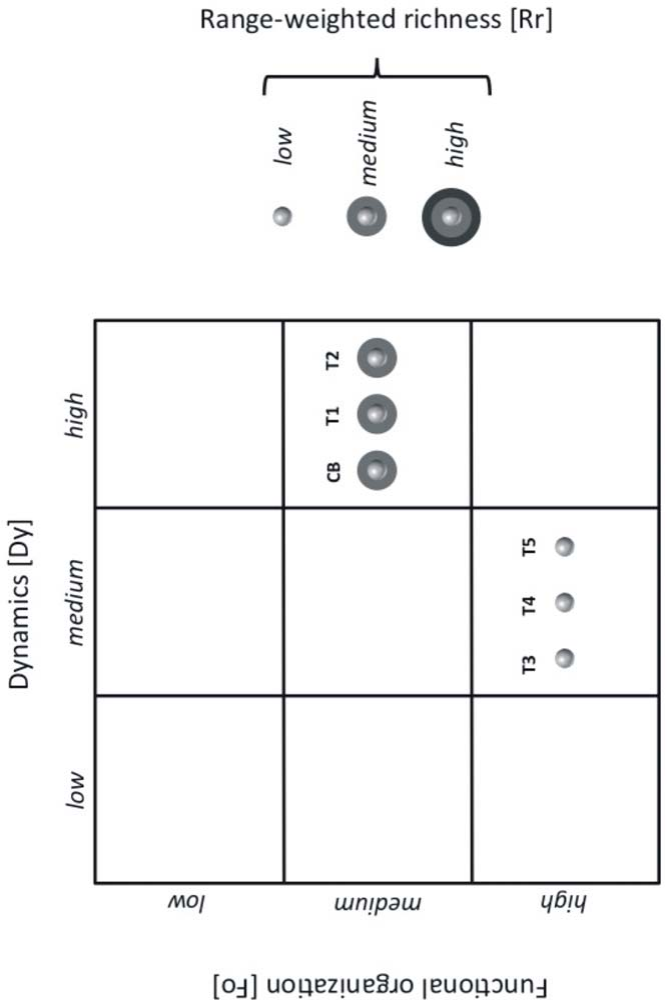
Schematic representation of DGGE results. The positioning of each sphere in a plot quadrate refers to a different ecological context and relative adaptation of the microbial community to the respective environmental conditions. CB control biofilm; T1–T5 biofilm exposed to increasing triclosan concentrations.

Comparative DGGE analyses of extracted DNA were carried out before and after TCS exposure to investigate possible shifts within the bacterial community. Biofilm without TCS served as a control to account for alteration of the community over time. Substantial differences in banding patterns of the TCS treated biofilms as compared to the controls revealed variations in the bacterial community composition and structure. The bacterial community diversity indices decreased along with increasing TCS concentrations: from 0.86 CB to 0.46 T5 (Simpson Diversity Index, from day 17) and from 2.15 CB to 0.65 T5 (Shannon-Weaver Index, from day 17). Thereby, the differences were most pronounced between control CB and lower TCS concentrations (T1 – T2: 20 – 100 µg/L) versus higher TCS concentrations (T3 – T5: 20 – 100 µg/L) (data not shown). A detailed analysis of the DGGE banding patterns following the approach described by Marzorati et al. [38] demonstrated considerable differences between the lower TCS concentrations (control CB and T1 – T2) and the higher TCS concentrations (T3 – T5). The analyzed data were plotted in a 2D graph with the projection of the range-weighted richness (Rr) values within a matrix of the calculated values for functional organization (Fo) and community dynamics (Dy) (Fig. 3).

The Rr value reflects the percentage of the gel that is covered by fingerprinting as well as the number of bands within that gradient section and thus reflects microbial diversity or the “carrying capacity” of an ecosystem [38]. With increasing triclosan concentrations, the average Rr values decreased from 14.5±2.2 (CB, T1 – T2) to 7.9±1.6 (T3 – T5). The dynamic value (Dy) represents the number of species becoming significant during a defined time interval [38]. All bacterial biofilms were relatively dynamic, indicated by a high number of different species (as represented by DGGE melting domains) becoming dominant and/or extinct within the community during the course of the experiment. These “rates of change” values were highest in the undisturbed biofilm CB (59.0) and higher for the lower TCS concentrations (T1 – T2: 42.5±2.5) as compared to the biofilms subjected to higher TCS concentrations (T3 – T5: 35.2±1.0). The functional organization (Fo) values expresses the relation between the structure of a microbial community and its functional redundancy. Marzorati et al. [38] defined ‘functional organization’ as the ability of a microbial community to form an adequate balance of dominant microorganisms and resilient ones. These conditions increase the likeliness that a microbial community can counteract the effect of a sudden stress exposure without loss of function. The calculated Fo values for the T3 to T5 bacterial biofilm were on average higher (59.8±2.9) than the Fo values calculated for the CB and T1 – T2 treatments (50.5±0.9). This might indicate the establishment of a highly specialized, low-diversity bacterial community at Triclosan concentrations >20 µg L^−1^.

Seven prominent DGGE bands from the control CB biofilm (1 band), the T1 (3 bands) and T2 biofilm (1 band), and the T5 biofilm (2 bands) were cut out, re-amplified, cloned, and sequenced (see Table S1). Based on their unique or ubiquitous appearance in the different treatments, the DGGE bands were categorized in four groups: (i) those excised only from the control biofilm with no TCS exposure; (ii) those that appeared in all DGGE patterns independent of TCS concentrations (representative bands cut out from T1 and T2); (iii) those that only were present at the lowest triclosan concentration (bands unique to sample T1); and (iv) those that only appeared at the highest triclosan concentration (bands unique to sample T5). For two bands only two clones could be successfully sequenced, while for all other bands four or five clones were retrieved. In total, we obtained 27 partial 16S rRNA gene sequences (read length 550 nucleotides): 4 sequences belonging to group one, 7 sequences belonging to group two, 6 sequences belonging to group three, and 10 sequences belonging to group four (see Table S1).

Sequence classification revealed that all separated DGGE bands consisted of multiple 16S rRNA gene sequences representing various phylotypes. This verified that the bacterial diversity of the biofilm was generally higher than the resolution power (band separation) of the DGGE. Nonetheless, some phylotypes were only associated with certain groups. For example, we found sequences belonging to the Bacteriodetes families *Porphyromonadaceae, Cryomorphaceae, Flavobacteriaceae*, and members of the Clostridiales family XI. (incertae sedis) only in the untreated control biofilm and up to triclosan concentration of 2 µg L^−1^ (T1). Sequences classified as *Brucellaceae* (Alphaproteobacteria) and *Carnobacteriaceae* (Firmicutes) were solely recovered from bands unique to triclosan concentration of 100 µg L^−1^ (T5). Invariant DGGE bands that occurred under all triclosan concentrations represented sequences belonging to the betaproteobacterial genus *Alcaligenes*.

### Changes in Colloidal EPS Components

**Figure 4:**
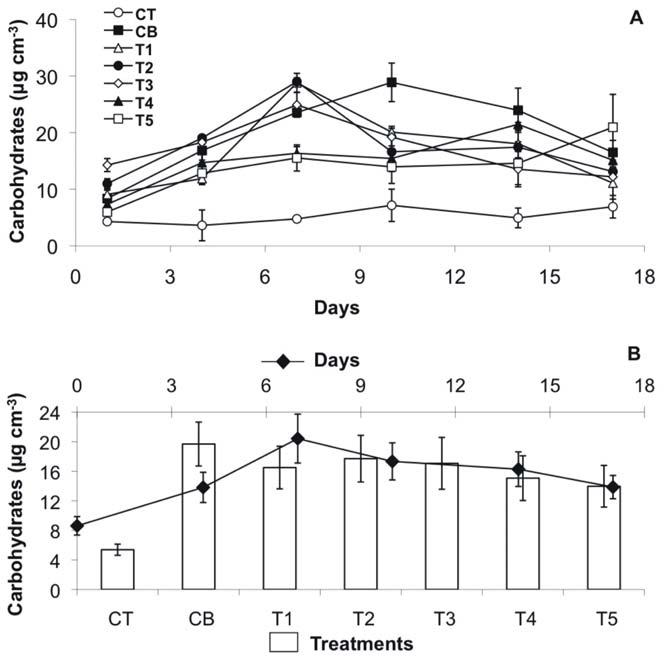
EPS carbohydrate concentrations over the course of the experiment. (A) Mean values (n = 4 per treatment, based on n = 3 replicates per box) with SE of the different treatments over time: positive control CB (black squares), negative control CT (white circles), T1 (TCS: 2 µg/L, white triangles), T2 (TCS: 10 µg/L, black circles), T3 (TCS: 20 µg/L, white diamonds), T4 (TCS: 50 µg/L, black triangle), T5 (TCS: 100 µg/L, white squares). (B) Mean values with SE per day (n = 7, black diamonds) and per treatment (n = 6, bar plots).

In the positive control and the treatments with low TCS concentrations, the colloidal EPS carbohydrate concentrations increased up to the middle of experiment and gradually decreased thereafter (Fig. 4 A, B). In contrast, treatments T4 and T5, with the highest TCS concentrations, showed a much lower increase over the first week, followed by an almost continuing increase until the end of the experiment. Thus, the final concentrations of EPS colloidal carbohydrates were similar between all treatments, except for T3 (Table 1). Averaged over the whole experiment, CB, T1 as well as T2 showed the highest carbohydrate concentrations as compared to the other treatments, with ranges between 8.35 – 28.9 µg cm^−3^, 9.09 – 28.8 µg cm^−3^, 11 – 29.01 µg cm^−3^, respectively (Fig. 4 B). For instance, on day 7, CB and T1 were significantly higher than T3, T4 and T5 (Permanova, p<0.0001, followed by a non-parametric SNK test). At the same time, T3 (range 14.27 – 24.9 µg cm^−3^) was significantly higher than T4 and T5 (range 7.34 – 21.5 µg cm^–3^ and 5.98 – 20.96 µg cm^–3^, respectively) (Permanova, p<0.0001, followed by a non-parametric SNK test) (Fig. 4 A). The negative controls without biofilms showed negligible concentrations of EPS carbohydrates.

**Figure 5:**
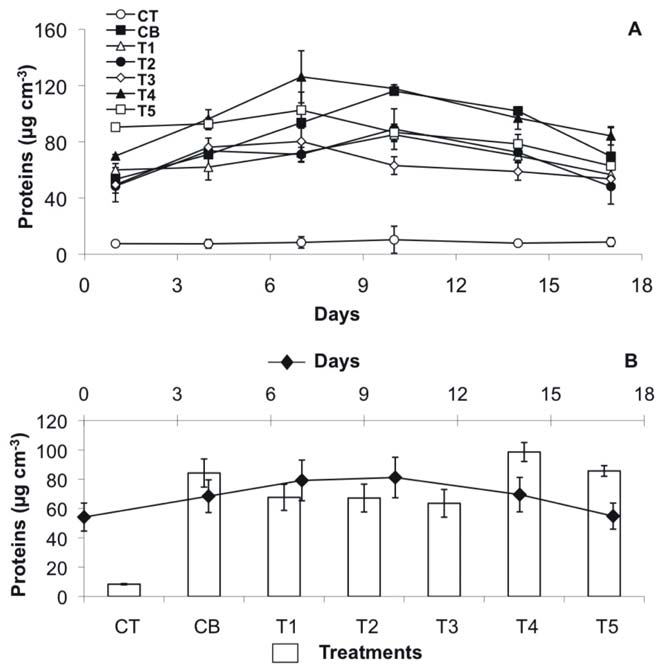
EPS protein concentrations over the course of the experiment. (A) Mean values (n = 4 per treatment, based on n = 3 replicates per box) with SE of the different treatments over time: positive control CB (black squares), negative control CT (white circles), T1 (TCS: 2 µg/L, white triangles), T2 (TCS: 10 µg/L, black circles), T3 (TCS: 20 µg/L, white diamonds), T4 (TCS: 50 µg/L, black triangle), T5 (TCS: 100 µg/L, white squares). (B) Mean values with SE per day (n = 7, black diamonds) and per treatment (n = 6, bar plots).

The water–extractable proteins showed a clear increase over the first half of the experiment and a decrease thereafter in all treatments (Fig. 5 A, B). However, the relative increase in EPS proteins from the start to the end of the experiment was most pronounced for the biofilm without TCS (up to 2.2 times, ranged between 53.3 – 116 µg cm^–3^, Table 1). Consequently, the positive control had significantly higher EPS protein concentrations on most of the sampling days as compared to T1 (range 60 – 85 µg cm^–3^), T2 (range 48.5 – 89 µg cm^–3^) and T3 (49.4 – 80.3 µg cm^–3^) (Permanova, p<0.0001, followed by a non-parametric SNK test Fig. 5 A). However, the treatments with the highest TCS concentrations (T4, T5) started with higher protein concentrations that were in a similar range to the positive control (between 69.9–126.2 µg cm^–3^ and 90.4–102.5 µg cm^–3^, respectively) (Fig. 5 B, Table 1). Accordingly, there were no significant differences between CB and T4 as well as T5.

A strong correlation was determined between EPS colloidal carbohydrates and EPS colloidal proteins for all treatments except T5 (n = 20 CB: r = 0.748; T1: r = 0.523; T2: r = 0.542; T3: r = 0.560; T4: r = 0.508; p<0.05).

### Relations between Biological Variables, Surface Adhesion and Triclosan Exposure

**Figure 6:**
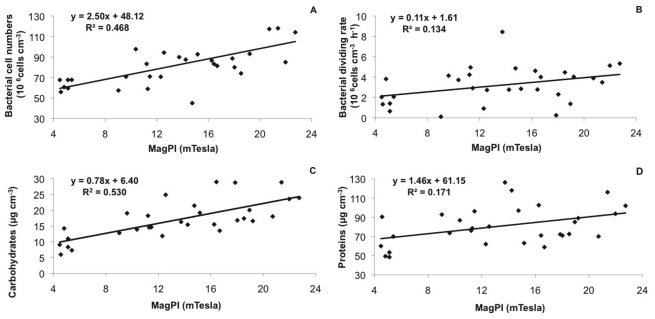
Scatter plot (n = 30) to show the relationship between bacterial biofilm adhesion expressed by MagPI (mTesla) versus bacterial cell numbers (A), bacterial division rates (B), EPS carbohydrate concentrations (C) and EPS protein concentrations (D).

Considering the complete dataset, positive relationships were found between substratum adhesion, bacterial cell numbers (Fig. 6 A) and bacterial division rates (Fig. 6 B). Furthermore, substratum adhesion was closely related to EPS colloidal carbohydrates (Fig. 6 C) and, to a lesser extend, to EPS proteins (Fig. 6 D). In the single treatments, the colloidal carbohydrates and proteins both showed significant relation to the bacterial division rates (e.g. CB: R^2^ = 0.834, p<0.01, for carbohydrates; CB: R^2^ = 0.590, p<0.05, for proteins) while the relation to the bacterial cell numbers were positive but non-significant. Taken together, the relationships became less strong and varied their significance. Focusing on the single treatments separately, the strongest correlations between and the biological parameters (bacteria, EPS) were generally determined for the treatments with no or lower triclosan exposure (Table 3).

**Figure 7:**
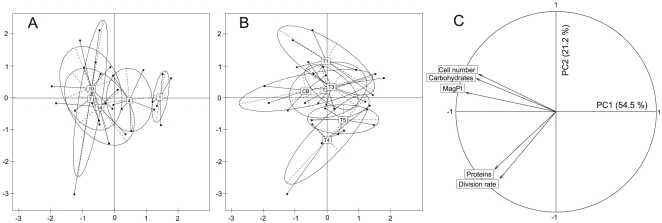
PCA. The projection of the objects in the plane formed by PC1 and PC2 showed that the gravity centers are distributed differently depending on whether they are grouped according to the sampling dates (A) or the treatments (B). (C) Circle of correlation for variables and projection of the variables in the factorial plane PC1 – PC2.

Principal component analysis (PCA) revealed that the first and second principal components (PC1 and PC2) explained about 75% of the total variability (inertia) (PC1: 54.5%, PC2: 21.2%). Treatments and sampling dates were grouped by computing the gravity center of each group together with an ellipse, which indicates the total variability of the group (i.e. width and height correspond to 1.5 times the eigen values of the corresponding covariance matrix). The PCA showed a separation of the gravity centers according to the sampling dates (Fig. 7 A) or the treatments (Fig. 7 B). Despite a relatively high variability within the groups (especially in Fig. 7 B), the gravity centers of the different sampling dates were clearly distributed along PC1 starting at the right end of the graph with the first days of biofilm growth towards the left end with the last days of the experiment (Fig. 7 A). Similarly, the gravity centers of the different treatments were distributed along PC2 with biofilms exposed to none or lowest triclosan concentrations located at the top and biofilms growing in the presence of highest TCS concentrations located at the bottom (Fig. 7 B).

**Table 3:** Pearson’s correlation coefficients between variables (surface adhesion (MagPI), EPS carbohydrates and proteins, bacterial cell numbers and bacterial dividing rates per treatment).

Treatment	*Carbohydrates*			*Proteins*			*Bacterial cell*			*Bacterial dividing rate*		
CB	0.774	20	**	0.795	20	**	0.528	20	*	0.834	13	**
T1	0.634	20	**	0.595	18	**	0.497	29	*	–0.154	14	
T2	0.542	16	*	0.548	20	*	0.537	16	*	0.626	12	*
T3	0.011	18		0.135	18		–0.233	18		0.094	12	
T4	0.667	20	**	0.483	20	*	0.438	16		0.642	12	*
T5	0.610	20	**	0.096	20		0.465	18	*	0.617	14	*

The significance levels are the following:

*** p<0.001.

** p<0.01.

* p<0.05.

In the second part of the PCA, the loadings were plotted within the correlation circle [39] (Fig. 7 C). Two groups of variables were identified: substratum adhesion (MagPI), EPS carbohydrates and bacterial cell numbers accounted for 29.8, 23.1 and 21.9%, respectively, of the PC1 variance (74.8% in total). The bacterial division rates and EPS proteins were in opposition to the first group and correlated to each other (Table 4). Although these two variables also contributed to PC1 (respectively 11.4% and 13.8%), they explained 42.1% and 31.0% (in total 73.1%) of the variability of PC2.

**Table 4:** Spearman’s rank correlation coefficient (ρ), N = 30, (p<0.001 = ***, p<0.01 = ** and p<0.05 = *).

	***MagPI***	***Carbohydrates***	***Proteins***	***Cell number***	***Dividing rate***
***MagPI***	1				
***Carbohydrates***	0.71***	1			
***Proteins***	0.36	0.26	1		
***Cell number***	0.70***	0.61***	0.31	1	
***Dividing rate***	0.39*	0.35	0.41*	0.21	1

Considering the scores and the loadings together, the multivariate analysis identified the increase of sediment stability, EPS carbohydrates and bacterial cell numbers with experimental time and their decrease along enhanced triclosan concentrations. Simultaneously, bacterial division rates and EPS proteins increased with time but also with increasing triclosan concentrations.

**Figure 8:**
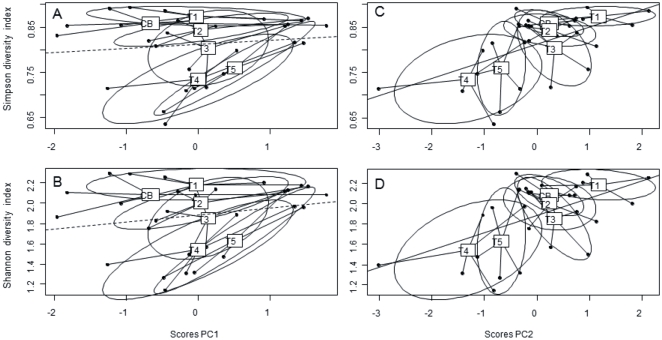
Linear regressions between bacterial diversity indexes and PC1 and PC2 scores of the Principal Component Analysis. Each treatment is represented by its ellipse inertia and its gravity centre (CB: control, T1–5: increasing triclosan concentration). Solid line: significant linear regressions, dashed lines: non-significant linear regressions.

Shannon-Wiener [40] and Simpson [41] Index were calculated based on the normalized DGGE banding patterns in GelCompar II to describe bacterial diversity. Both diversity indices were plotted against PC1 and PC2 scores to identify relationships between bacterial diversity and biofilm development/stability in dependence of time (PC1) and triclosan exposure (PC2) (Fig. 8). The ellipses inertia of each treatment along with their gravity centres did not reveal significant relations between the Shannon or Simpson diversity index and PC1 scores (Fig. 8 A, C, ρ = 0.08 and 0.20 respectively, p>0.05). In contrast, significant relationships were determined between bacterial diversity and PC2 scores (Fig. 8B, D, ρ = 0.53 and 0.41, p<0.05 and 0.01 respectively). Thus, the bacterial diversity, as represented by species richness and species evenness, was decreasing with enhanced triclosan concentrations (Fig. 8 B, D).

## Discussion

### From Bacterial Attachment to Substratum Stabilization – Observed Effects of Triclosan

This is the first study to investigate the effect of triclosan (TCS) on the stabilization potential of bacterial biofilms inhabiting sediments in aquatic environments. The TCS concentrations chosen were of environmental relevance in the lower range. Although the medium and higher TCS concentrations were deemed much higher than the data measured presently in the waters of the aquatic habitats, they are within the accumulation rates of TCS determined in the sediment. Moreover, the choice accounted for the continuous replenishment of TCS by our modern lifestyle that might lead to significantly rising TCS concentrations in the future.

**Figure 9:**
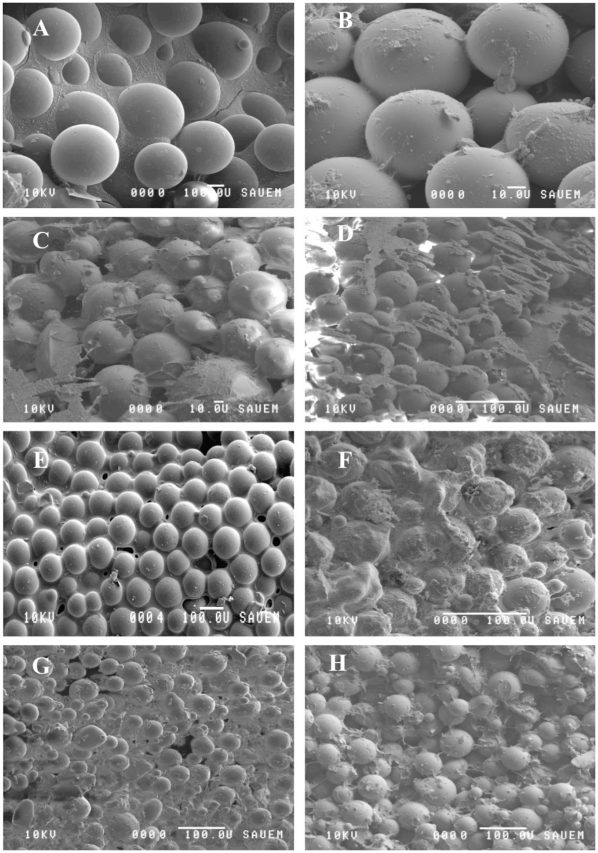
Low-temperature scanning electron microscopy (LTSEM) images of the biofilms: A B: controls (negative and positive, higher magnification) at day 1 C D: T 2 and T5 at day 1 E F: controls (negative and positive, lower magnification) at day 7 G H: T2 and T5 at day 7. A/E: Water frozen around the clean glass beads of the control without biofilm. In the presence of bacteria (B – D; F – H), a matrix of EPS is visible, heavily covering the glass beads and permeating the intermediate space. However, at day 7, the biofilm exposed to higher TCS concentrations (H) showed a visibly less dense EPS matrix as compared to the low TCS concentration treatment (F).

Initial bacterial colonization significantly stabilized the test substratum. Since the chosen substratum was composed of non-cohesive glass beads, the binding force must have been entirely due to bacterial attachment and the secretion of a polymeric matrix (Fig. 9) [34]. In contrast, the negative control (CT) did not show any variations in substratum stability over time. The stabilization effect was significantly more pronounced for the positive control CB without TCS, than for the treatments with TCS exposure and was over 5 times higher than negative control CT. The impairment of the bacterial stabilization was significantly more pronounced along the increasing TCS gradient. However, even the highest TCS concentrations did not prevent bacterial settlement and biofilm development since the overall stability increased initially over time in all treatments. However, the “slopes of increase” were lower in the TCS treatments as compared to the control CB, especially at the beginning of the incubations. The data suggested that TCS interfered with the initial adhesive properties of the biofilm as it was described under the exposure to selected pharmaceuticals by Schreiber and Szewzyk [42]. After only one week, the stability of the biofilm exposed to the highest TCS concentration (T5: 100 µg L^–1^) decreased significantly; the same effect was observed much later (day 14 – day 17) in the other treatments (CB, T1 – T4: 2 – 50 µg L^–1^). In former experiments, without a continuous nutrient supply, decreasing microbial substratum stabilization was observed after time and deemed as a typical “batch culture effect” caused when the initial culture nutrients have been used up [32], [34]. In the present experiment, the exposure to TCS seemed to have additionally impeded the stabilization potential in nutrient depleted cultures. This is in contrast to the findings of Johnson et al. [43] who reported on an enhanced sensitivity of bacteria to TCS in the presence of ample nutrients [43].

### The Significant Role of the EPS Matrix for Binding and the Influence of Triclosan

In recent years it has been highlighted that microbial EPS (extracellular polymeric substances) may significantly stabilize the sediment [31], [44]. While the focus in biostabilization research has long been on polysaccharides, proteins are an abundant part of the EPS [45]; thus carbohydrates and proteins were analyzed in the present samples. In fact, the increasing surface adhesion was mirrored by increasing EPS concentrations for both carbohydrates and proteins in the first week of the experiment in all treatments. After seven days, the EPS levels dropped most in the biofilm exposed to high TCS levels (>20 µg L^–1^), followed later by the positive control and (for proteins) by treatments with low TCS concentrations (<10 µg L^–1^). Thus in most cases, there was a time lag between decreasing EPS levels and the loss of the adhesive capacity and stabilization by the biofilm, which occurred after the second week (except for T5 with parallel decrease of EPS and stability). The overall correlations between EPS concentration and substratum stability were highly significant (stability - carbohydrates r = 0.728, n = 29, p<0.001; stability - proteins r = 0.414, n = 29, p<0.05); giving evidence of the important role of EPS for both, developing and reversing substratum stability.

The bacterial biofilm under TCS exposure did not show elevated EPS levels as might have been expected, especially at the beginning on the incubation, as a possible defense strategy of the microbes to create a barrier between cell and toxicant [35]. On the contrary, the EPS carbohydrate concentrations were significantly highest in the positive control, followed by the biofilm exposed to low and medium (<20 µg L^–1^) TCS levels and significantly lowest in the treatments with high TCS exposure (50 – 100 µg L^–1^). This pattern was reflected by significantly lower bacterial cell numbers in the T4 and T5 treatments as compared to the CB and T1, but also to T2 – T3 over the course of the experiment. This corroborates earlier findings on TCS effects (concentration 10^–3^, 10^–4^, 10^–5^M) on the density of bacteria in biofilms [46]. The data suggested a primary impact on bacterial metabolism and reproduction by TCS exposure which subsequently affects EPS secretion, as suggested by Onbasli and Aslim [36]. The strong relation between bacterial growth and EPS carbohydrate concentrations underlines this.

The pattern was quite similar for the EPS proteins, except for the elevated protein levels in the T4 and T5 treatments. It has been indicated that TCS acts as a bacteriostatic agent at low concentrations, inhibiting bacterial growth and reproduction [16], [23], but becomes bactericidal at higher concentrations causing permanent damage to the bacterial membrane [24]. For instance, Ricart et al. [27] reported on steeply decreasing live/dead ratio of bacteria with increasing TCS concentrations (e.g. 0.7 in control as opposed to 0.3 for 50 – 100 µg L^–1^) and environmental relevant concentrations caused increased mortality (No Effect Concentration (NEC) of 0.21 µg L^–1^). Thus, it can be assumed that the higher TCS concentrations in the present experiment (>50 µg L^–1^) induced bacterial cell lysis with a consequent release and augmentation of intracellular components such as proteins. This type of protein did not apparently contribute to any binding or adhesion effects since substratum stabilization was significantly lowest in the T4 and T5 treatment.

It has been stated before that EPS quantity and also EPS composition (“quality”) is decisive for the microbial binding effect [32], [33], [34]. There is increasing evidence that proteins of hydrophobic character seem to play a significant role in the first adhesion of bacteria as well as contribute towards the binding strength within the developing EPS matrix [47], [48]. This is in contrast to the earlier opinions that these EPS proteins were solely extracellular enzymes to prepare exterior macromolecules for the bacterial cell uptake, it has since became apparent that proteins also have structural significance [49]. In the present experiment, apart from the presumably intracellular protein levels in T4 and T5, both EPS components, carbohydrates and proteins, were always significantly correlated to substratum stability. It is suggested, that these interactions between carbohydrates and proteins are important for the observed binding effects [33], [34], [49]. Future studies should relate EPS composition and quantity to the adsorption capacity of the biofilm matrix which would additionally reduce the bioavailability and toxicity of pollutants. The data reveal a similar response of carbohydrate and protein EPS components because of bacterial growth impairment due to TCS exposure, to influence substratum stabilization. These effects of TCS or other toxicants/pollutants on an important functionality of microbial systems (biostabilization), have to our knowledge never been shown before.

### Bacterial Diversity and Community Composition Under Triclosan Exposure

EPS secretion (and thus quantity and quality) and substratum stabilization are not only influenced by the biomass or cell number of the microbial producer, but also by their physiological state and their community composition [50]. The diversity of a microbial community largely determines their resilience to fluctuating biotic and abiotic conditions, including toxicant exposure, and thus, their ongoing functional capability [51]. In the present experiment, the Simpson’s diversity index (1 – D) as well as the Shannon Wiener index (both calculated from the DGGE banding patterns) indicated a highly diverse bacterial community in the control biofilm and decreasing diversity with increasing TCS exposure. The functional organization (Fo) also reflected the establishment of a highly specialized low-diversity microbial community at high TCS concentrations above 20 µg L^–1^. The significantly lower range-weighted richness (Rr) values <10 determined in the TCS exposed treatments can be attributed to environments particularly adverse or restricted to colonization such as areas exposed to chemical stress [38]. Similarly, the dynamic values (Dy) indicated a lower rate of change, especially in T3 – T5 that might reflect enhanced detachment and biofilm dissolution in the presence of high concentrations of the broad-spectrum antibacterial compound TCS. The observed decrease in DGGE band pattern complexity with increasing triclosan concentrations were mirrored by a decrease in EPS quantity and biostabilization. Previous literature values reported for microbial communities exposed to chemical stress conditions in diverse, highly dynamic ecosystems such as those found in silage fermentation and activated sludge matched the results of the present study [52], [53].

Along with the changes in bacterial diversity, there was a pronounced shift in species composition with increasing exposure to TCS. While members of the phylum Bacteriodetes (Porphyromonadaceae/*Proteiniphilum acetatigenes*, Cryomorphaceae /*Brumimicrobium glaciale*, Flavobacteriaceae/*Salegentibacter mishustinae*) were always present within the control biofilm and at low TCS concentrations, they were not detectable under higher TCS exposure, indicating the strong sensitivity of the species detected to triclosan. TCS inhibits fatty acid synthesis with subsequent perturbation of the bacterial membrane [13] but it also interferes with the quorum-sensing signaling of Gram-negative bacteria; thus inhibiting their attachment, growth and formation of biofilm [46]. In that context, Dobretsov et al. [46] reported on the specific sensitivity of Alpha- and Gammaproteobacteria as well as on Cytophagia of the phylum Bacteroidetes to concentrations of TCS of 10^–3^ M in contrast to the unaffected Gram-positive phylum of Firmicutes. While in the present experiment Bacteroidetes members were indeed sensitive to TCS, species belonging to the Alphaproteobacteria (Brucellacea/*Pseudochrobactrum glaciei*) as well as Firmicutes (Carnobacteriaceae/*Carnobacterium mobile, C. inhibens*) were found solely from samples exposed to high TCS concentrations (T5, 100 µg L^–1^). Thus, we suggest species may tolerate elevated TCS levels either through effective detoxification mechanisms (e.g. active efflux from the cell), the ability to biodegrade/inactivate TCS (e.g. expression of TCS degrading enzymes) or to develop resistance to TCS (e.g. mutations in the enoyl reductase) [17], [18]. The inconsistent results as compared to the literature might be due to the fact that phylotypes of the same class, order, family or even species can vary substantially in their sensitivity to pollutants such as triclosan, from being completely resistant to susceptible. Hence, the results on adaptation or sensitivity versus triclosan presented here are not to be generalized for the whole taxa.

Invariant DGGE bands occurring in samples of all treatments belonged to the betaproteobacterial genus Alcaligenes/*Alcaligenis faecales*. Betaproteobacteria seem to be of more widespread occurrence and general importance in freshwater habitats than marine habitats [54]. Brümmer et al. [55] allocated similar bands/clusters of Betaproteobacteria to biofilms within the Elbe River and its polluted tributary the Spittelwasser River.

In conclusions, the diversity and species composition of bacterial assemblages was impaired by TCS exposure in the present experiment, but these effects were most pronounced at higher TCS concentrations (T3 – T5). Lawrence et al. [22] reported significantly different DGGE patterns in biofilms exposed to environmentally relevant TCS concentrations while there were little variations in the bacterial community in the present study below 10 µg L^–1^ TCS. In general, shifts in community structure due to TCS exposure do not necessarily imply changes in the functionality of these communities [56]. However, in the present study, even small shifts in the bacterial assemblages at low TCS concentrations resulted in a significantly impact on EPS secretion and related influence on the stabilization potential. Despite the development of a rather specialized community, the bacterial biofilms in our batch cultures could not recover full functionality in terms of biostabilization during the time of the experiment, even at the lowest TCS concentration. Theoretically, the conservation of a given functionality is often ensured by the flexibility of a microbial community with minority community members that may become dominant in a short period following significant perturbation; in this way functional redundancy can assure fast recovery from a stress condition such as exposure to toxic chemicals [38]. It remains open to debate whether a natural biofilm composed of bacteria, microalgae and protozoa, continuously supplied by nutrients, would be able to adapt to increasing triclosan concentrations over time. Thee impairment of biostabilization has already been shown for TCS concentrations that are currently been measured in the river waters (around 2 µg L^–1^). Yet, the TCS concentrations accumulating in the natural sediments are much higher, continuously increasing and of true relevance for sedimentary biofilms. Thus, the applied higher triclosan levels in the present study are of significance for the sediment habitats and provide a warning in terms of possible effects to consider in the future.

Biostabilization is an important function for the aquatic habitat due to its impact on the dynamics of sediments and related microbial activity. Sediment erosion and transport is indeed critical to the ecological (e.g. bioavailability of associated pollutants), social (e.g. clean drinking water) and commercial (e.g. sediment dredging from harbours, coastal erosion) health of aquatic habitats from watershed to sea. Hence, microbial sediment stabilization can be regarded as one significant ecosystem service.

## Conclusions

In the present experiment, TCS exposure affected the growth and physiology of a bacterial biofilm and resulted in varying EPS patterns that impaired their substratum stabilization potential, one important ecosystem function. However, it remains unknown if the observed shifts in species composition and diversity were affecting other biofilm functions (e.g. adsorption capacity and degradation potential for pollutants within the biofilm matrix). Future studies should be expanded to relate multiple functional attributes to selected bacterial species and assemblages to investigate the functional significance of species shifts and environmental challenges such as xenobiotic compound and other environmental stress.

## Materials and Methods

No specific permits were required for the isolation of the bacteria from the field and the described laboratory studies. The location is not privately-owned or protected in any way. The field studies did not involve endangered or protected species.

### Bacterial Cultures

Sediment was sampled to a depth of 5 – 10 mm from a mudflat in the intertidal of the Eden estuary located in the southeast of Scotland (56°22′N, 2°51′W). The sediment was mixed with 1 µm-filtered seawater (1:1) and the sediment slurry was sonicated (Ultrasonic bath XB2 50–60Hz) for 5 min to enhance detachment of bacteria from the sediment grains. After centrifugation (2 times, 10 minutes, 6030 g, Mistral 3000E, Sanyo, rotor 43122-105) to remove the sediment, the supernatant (bacteria) was transferred and centrifuged once again (10 minutes, 17700 g, Sorval RC5B/C). This time the supernatant was discarded, while the remaining pellet with the majority of bacteria was resuspended and filtered through a 1.6 µm filter (glass microfiber filter, Fisherbrand MF100) to separate bacteria from benthic microalgae (smallest expected size from the Eden estuary: 4 – 10 µm). The bacteria were cultivated for 3 weeks in acid-washed 200 ml Erlenmeyer flasks under constant aeration in the dark, at room temperature (15°C) and supplied regularly by autoclaved standard nutrient broth (1 : 3; Fluka, Peptone 15 g/L, yeast extract 3 g/L, sodium chloride 6 g/L, D(+)glucose 1 g/L). Microalgal contamination was checked regularly by epifluorescense microscopy.

### Experimental Set-up and Triclosan Spiking

Since triclosan (TCS) is of highly absorptive character, the use of plastic boxes had to be avoided. Thus, small glass tanks were used (in mm 105L×105W×55H) in which a 1 cm layer of <63 µm glass beads was prepared as non-cohesive substratum for biofilm growth. The boxes were gently filled with 300 ml of autoclaved seawater (controls) that has been spiked with defined TCS concentrations (treatments). For the latter, the stock solution of TCS was prepared by dissolving the commercial available powder (Irgasan, Sigma-Aldrich C.N 72779) in seawater with the help of a magnetic stirrer (STUART GB) for four hours. The stock solution was diluted with seawater to gain the defined concentrations of 2 µg/L, 10 µg/L, 20 µg/L, 50 µg/L, and 100 µg/L of triclosan. Except for the negative control, the glass boxes were further inoculated by 10 ml of bacterial stock solution to initiate biofilm growth.

The following treatments were established each with four replicates:

1. bacterial culture + 2 µg/L of triclosan (T1)
2. bacterial culture + 10 µg/L of triclosan (T2)
3. bacterial culture + 20 µg/L of triclosan (T3)
4. bacterial culture + 50 µg/L of triclosan (T4)
5. bacterial culture + 100 µg/L of triclosan (T5)
6. negative control (CT): no triclosan, no bacterial culture
7. positive control (CB): no triclosan, plus bacterial culture

The negative control (CT), containing only glass beads and seawater, was treated once a week with a mixture of antibiotics (150 mg/L streptomycin and 20 mg/L chloramphenicol, final concentrations) to prevent bacterial colonisation. All treatments were gently aerated and kept at constant temperature (15°C) in the dark, over the experimental period of 2 weeks.

### Sampling

Sampling took place every second day during the experiment. For each replicate (four) of the treatments and the controls, four cores of substratum (2 mm depth) were removed using a cut-off syringe (10 mm diameter). The cores were immediately processed for the determination of bacterial cell numbers and dividing rates or frozen at –80°C for further analysis of extracellular polymeric substances (EPS) and DNA extractions for bacterial community analysis. To monitor triclosan concentrations over time, samples of water and substratum (additional cores of 5 mm depth) were taken at the beginning (sampling day 1), in the middle (sampling day 4) and at the end of the experiment (sampling day 7) from each box. Thereby, four cores per treatment were pooled within a 15 ml Apex centrifuge tube to account for spatial heterogeneity and stored for future analysis at –80°C.

### Bacterial Enumeration by Flow Cytometry

Cores for bacterial cell counts were fixed with glutaraldehyde (1% final concentration) and bacteria were stained with Syto13 (Molecular Probes, 1∶2000 v:v, 1.2 µmol/L final concentration) for 15 min in the dark. The flow rate of the flow cytometer (Becton Dickinson FACScan™ with a laser emitting at 488 nm) was fixed to 60 µl/min and the data were recorded until 10000 events were acquired and/or 1 minute had passed. Bacteria were detected by plotting the side light scatter (SSC) versus green fluorescence (FL1). An internal standard was added to some samples (PeakFlow™ reference beads, 6 µm size, 515 nm, Molecular Probes) to distinguish bacterial cells from debris and mineral particles. The data were analyzed using the “Cellquest” software. Bacterial cell numbers are given as content in cells per cm^–3^ of sediment.

### Bacterial Division Rate

Immediately after sampling, the cores (triplicates) were incubated for 20 min with [*methyl-* ^3^H] thymidine (final concentration 300 nmol/L, S.A., 50 Ci mmol^–1^ [57], [58] until the incorporation of radioactive thymidine was stopped by adding 5 mL of 80% ethanol [59]. Afterwards, the samples were collected on a filter (0.2 µm), washed several times with 80% ethanol and 5% ice-cold trichloroacetic acid (TCA) and mixed with 5 mL of 0.5 mol/L HCl and incubated at 95°C over 16 hours [60]. For further details please see Lubarsky et al. [33]. A subsample of the supernatant was finally mixed with 3 mL of the scintillation cocktail Ultima Gold MV. The bacterial division rate (cells cm^–3^ h^–1^) was calculated by the internal standard quenching curve (Liquid scintillation analyzer “TRI-CARB 2000”) while assuming that 1 mol^–1^ incorporated thymidine equivalents the production of 2×10^18^ bacterial cells [61], [62]. The data have been corrected by a blank (mean of two replicates) that corresponds to pre-fixed sediment cores submitted to the protocol described above. Bacterial dividing rate are given as content (10 ^6^ cells cm^–3^ h^–1^).

### Bacterial Community Analysis by Denaturing Gradient Gel Electrophoresis (DGGE)

The bacterial community has been monitored before and after the TCS exposure and compared to the control (biofilm without TCS) to distinguish between bacterial community shifts due to TCS exposure and time. Total DNA was extracted from 0.25 g of the frozen cores using the Ultra Clean DNA Soil Extraction kit (MoBio Laboratories, Carsbad, CA) according to the manufacturer’s instructions. The extracted DNA was used as template in PCR reactions in order to amplify a fragment of the bacterial 16S rRNA gene using ‘universal’ primers. The forward primer was the one previously published by Muyzer et al. [63] (341-F-GC). As reverse primer a modified version of the primer sequence published by Muyzer and Ramsing [64] (907R-mod. 5′-CCGTCAATTCMTTTRAGTTT-3′) has been used [64]. For DGGE the forward primer was preceded by a 40 nucleotide GC-clamp [63]. PCR amplification was conducted in a 50 µL reaction containing 100 ng of template DNA, 10 pmol of each primer, 1.25 U of Taq DNA polymerase (Go Taq, Promega), 1 × PCR buffer, 3.5 mM MgCl_2_, and 200 µM dNTPs. Amplification was performed in a MyCycler thermal cycler (BIO-RAD Laboratories, Munich, Germany) with the following touchdown program: Initial denaturation 94°C for 3 min, followed by 20 cycles of denaturation at 94°C for 1 min, annealing at 65°C (decreasing each cycle by 0.5°C) for 1 min and an elongation step at 72°C for 1 min. Following these steps, another 12 cycles of 94°C for 1 min, annealing at 55°C for 1 min, and elongation at 72°C for 1 min, with a final elongation step at 72°C for 9 min, was performed. Product amplification was verified by electrophoresis on a 1.5% (w/v) agarose gel stained with ethidium bromide.

DGGE of the PCR products was performed on a 6% (w/v) polyacrylamide gel with urea and formamide as denaturants. The denaturing gradient was between 35% and 65% (100% denaturant contained 7 M urea and 40% deionized formamide). Electrophoresis was performed in 1 × Tris-acetate EDTA (TAE) buffer [40 mM Tris, 20 mM acetic acid, and 1 mM EDTA] at 60°C at constant voltage of 100 V for 18 h. Subsequently, gels were silver stained according to the protocol of Bassam et al. [65]. Stained gels were imaged on a UV/VIS converter plate using the Bio-Vision 3000 gel documentation system and software (Vilber Lourmat, Eberhardzell, Germany). Gel images were then analyzed using the GelCompar II software package (Applied Math, Kreistaat, Belgium). Calculation of diversity indices (Shannon, Simpson) was done within GelCompar II using the respective plug-ins. Interpretation of the 16S rRNA gene molecular fingerprinting pattern was performed according to the concept suggested by Marzorati et al. [38] including processing of range-weighted richness, dynamics and functional organization. DGGE bands of interest were cut from ethidium bromide stained gels and re-amplified in a PCR reaction (as described above) using the ‘universal’ DGGE primer without GC clamp. The TOPO TA Cloning® kit (Invitrogen Inc. Carlsbad, CA) was used to clone the re-amplified DGGE bands (pCR® 4-TOPO® vector and One Shot Chemically Competent *E. coli* cells). The maximum amount of DNA (4 µl DNA in Tris-buffer (10 mM), pH 8) was used in each of the cloning reactions following the manufacturer’s instructions. Three clones per band were selected and grown overnight in 5 mL LB broth containing 100 µg/mL ampicillin. The peqGOLD Plasmid Mini Kit I (PEQLAB Biotechnology GMBH, Erlangen, Germany) was used to purify plasmid DNA from 2 mL of the overnight culture. Plasmid DNA was send to GATC Biotech AG (Constance, Germany) for sequencing of the inserts (cloned DGGE bands) using the flanking vector primers M13 forward and reverse. Obtained sequences were manually trimmed and edited in Geneious Pro 4.7 (Biomatters ltd., Auckland, New Zealand) and aligned using the SINA aligner of the ARB software package (v 5.2) [66], [67] and the corresponding SILVA SSU Ref 102 database [68]. Sequence classification was done in Mothur v.1.13.0. using the SINA alinment and the SLIVA taxonomy [69].

### Nucleotide Sequence Accession Numbers

The partial 16S rRNA gene sequences from this study have been submitted to EMBL and assigned accession numbers FR850103 to FR850129.

### EPS Extraction and Determination

The sediment cores were mixed with 2 mL of distilled water and continuously rotated for 1.5 h by a horizontal mixer (Denley Spiramix 5) to extract the loosely-bound fraction of EPS at room temperature (20°C). After centrifugation (6030 g, 10 minutes, Mistral 3000E Sanyo, rotor 43122-105) the supernatant containing the water-extractable (colloidal) EPS fraction was pipetted into new Eppendorfs to analyze carbohydrates and proteins in triplicates following the Phenol Assay protocol [70] and the modified Lowry procedure [71], respectively. The adsorption for EPS carbohydrates and proteins was read by a spectrophotometer (CECIL CE3021) at the wavelengths 488 nm and 750 nm and calibrated versus defined concentration ranges (0 – 200 µg/L) of glucose and bovine albumin, respectively. For more details please see [32], [34]. The EPS carbohydrates and proteins concentrations are given in microgram per cubic centimeter (µg cm^–3^).

### Magnetic Particle Induction (MagPI) Measurements

This new method is based on the magnetic re-capturing of ferromagnetic fluorescent particles (Partrac Ltd, UK, 180 – 250 µm) that have been spread onto a defined area of the substratum/biofilm surface. The force of the overlaying electromagnet (magnetic flux) needed to retrieve the particles is a highly sensitive measure of the retentive capacity of the substratum, a proxy for adhesion. The electromagnetic force applied is accurately controlled by a precision power supply (Rapid 5000 variable power supply) and the particle movements are precisely monitored at each increment of voltage/current. The MagPI (Magnetic Particle Induction [37]) was calibrated using a Hall probe and the results are given in mTesla. The MagPI has been successfully used in a number of experiments and showed good correlations with the CSM (Cohesive Strength Meter), a well-established erosion device [32], [33].

### Determination of Triclosan Concentration

To investigate the effects of triclosan on bacterial biofilm growth at the substratum/water interface, the treatments were spiked via the water phase. Consequently, the actual triclosan (TCS) concentrations and distribution between the water phase and the surface substratum were regularly analyzed during the experiment by high performance liquid chromatography (HPLC). Before analysis, the extracts of the pooled cores (4 for each treatment), were obtained by careful separation of the overlaying water from the sediment using 20 mL syringe. The water samples and the extracts of the substrata were pre-concentrated using silica-based octadecyl bonded phase cartridges C18 6cc (SPEs) (Oasis HLB, Waters, Milford, MA), used to adsorb molecules of weak hydrophobicity from aqueous solutions. Prior to use, the SPEs cartridges columns (3 mL) were activated and conditioned with 5 mL of HPLC water, acetone and finally, methanol, at a flow rate of 1 mL/min. Samples (13 ml each) were promptly loaded onto the SPEs cartridges at a flow rate of 5 mL/min to avoid any degradation of the target compounds and the loss of sample integrity. After pre-concentration, the SPEs were completely dried by vacuum for about 20 min to avoid hydrolysis and kept at –20°C until analysis. Finally, the cartridges were eluted with 2 mL of methanol and directly injected onto the HPLC vials. The HPLC system consisted of a Waters 717 autosampler and a Waters 1525 binary pump. Separation of the compounds due to different polarity was achieved on a 5 µm, 150×4 mm i.d. C18 reversed-phase column (SunFire, Waters, Milford, US). The injection volume was set at 100 µL, and the flow rate was kept at 1 mL/min of 80% methanol using isocratic flow. Detection of TCS was carried out by a UV-VIS detector (Waters 2489) at the wavelength of 280 nm. The TCS peak was quantified against an absolute standard (Sigma-Aldrich, St. Louis, MO, highest purity, dissolved in methanol to 1 mg/L) using Empower 2 Chromatography Software (Waters). All solvents and standards used were of the highest purity available (HPLC grade, Sigma-Aldrich). Triclosan concentrations are given in microgram per litre (µg/L).

### Statistics

The data did not meet the assumptions required for ANOVA: none of the variables tested were normally distributed although equality of variance was verified for most of them (Shapiro normality test and Bartlett test for homogeneity of variance). Thus, differences between treatments were addressed using a permutational univariate analysis of variance (Permanova, 999 permutations) with R©2.9.0 (package “vegan” [72] followed by a non-parametric post-hoc Student-Newman-Keuls (SNK) test to compare pairs of treatments.

All the measured variables were analyzed by Principal Component Analysis (PCA) with R©2.9.0 (package “ade4” [73]). Briefly, eigen value decomposition of a data covariance matrix was performed from a dataset containing the following variables: colloidal EPS (proteins and carbohydrates), bacterial cell numbers, bacterial division rates and substratum adhesion (MagPI). The aim of the decomposition was to generate principal components (PC1 and PC2) that explain the majority of the total variance of the whole dataset. The calculation was performed with centred and scaled values after deleting rows that contained missing values. Scores were then plotted twice, clustered according to either the treatment name or the sampling date (objects). Loadings were visualized in the correlation circle. Both, scores and loadings were plotted separately for a better readability. Additionally, PC1 and PC2 scores generated by the PCA were plotted against bacterial diversity indexes (Shannon and Simpson).

## Supporting Information

Table S1 **Partial 16S rRNA gene sequences obtained from DGGE bands.** The sequences (27 partial 16S rRNA gene sequences in total, read length 550 nucleotides) recovered from DGGE bands were categorized as: (group 1) those excised only from the control biofilm with no TCS exposure; (group 2) those that appeared in all DGGE patterns independent of TCS concentrations (representative bands cut out from T1 and T2); (group 3) those that only were present at the lowest triclosan concentration (bands unique to sample T1); and (group 4) those that only appeared at the highest triclosan concentration (bands unique to sample T5).
(XLS)
